# Deep Sequencing of Plant and Animal DNA Contained within Traditional Chinese Medicines Reveals Legality Issues and Health Safety Concerns

**DOI:** 10.1371/journal.pgen.1002657

**Published:** 2012-04-12

**Authors:** Megan L. Coghlan, James Haile, Jayne Houston, Dáithí C. Murray, Nicole E. White, Paula Moolhuijzen, Matthew I. Bellgard, Michael Bunce

**Affiliations:** 1 Australian Wildlife Forensic Services and Ancient DNA Laboratory, School of Biological Sciences and Biotechnology, Murdoch University, Murdoch, Australia; 2 Centre for Comparative Genomics, Murdoch University, Murdoch, Australia; American Museum of Natural History, United States of America

## Abstract

Traditional Chinese medicine (TCM) has been practiced for thousands of years, but only within the last few decades has its use become more widespread outside of Asia. Concerns continue to be raised about the efficacy, legality, and safety of many popular complementary alternative medicines, including TCMs. Ingredients of some TCMs are known to include derivatives of endangered, trade-restricted species of plants and animals, and therefore contravene the Convention on International Trade in Endangered Species (CITES) legislation. Chromatographic studies have detected the presence of heavy metals and plant toxins within some TCMs, and there are numerous cases of adverse reactions. It is in the interests of both biodiversity conservation and public safety that techniques are developed to screen medicinals like TCMs. Targeting both the p-loop region of the plastid *trnL* gene and the mitochondrial 16S ribosomal RNA gene, over 49,000 amplicon sequence reads were generated from 15 TCM samples presented in the form of powders, tablets, capsules, bile flakes, and herbal teas. Here we show that second-generation, high-throughput sequencing (HTS) of DNA represents an effective means to genetically audit organic ingredients within complex TCMs. Comparison of DNA sequence data to reference databases revealed the presence of 68 different plant families and included genera, such as *Ephedra* and *Asarum*, that are potentially toxic. Similarly, animal families were identified that include genera that are classified as vulnerable, endangered, or critically endangered, including Asiatic black bear (*Ursus thibetanus*) and Saiga antelope (*Saiga tatarica*). Bovidae, Cervidae, and Bufonidae DNA were also detected in many of the TCM samples and were rarely declared on the product packaging. This study demonstrates that deep sequencing via HTS is an efficient and cost-effective way to audit highly processed TCM products and will assist in monitoring their legality and safety especially when plant reference databases become better established.

## Author Summary

Chemicals derived from plants and animals are widely used in traditional Chinese medicine (TCM), and it is commonplace for remedies to contain a complex list of ingredients. Due to their heterogeneous origins, and subsequent processing into pills and powders, it can be difficult for the biological origin of ingredients within each remedy to be reliably determined. In this study, we have, for the first time, used a second-generation DNA sequencing method to analyse TCM remedies and determine their animal and plant composition. Using this deep-sequencing approach we identified plant species that are known to contain toxic chemicals and identified animal DNA from species that are currently endangered and protected by international laws. Consumers need to be made aware of legal and health safety issues that surround TCMs before adopting them as a treatment option. More widespread testing of complementary medicines using the DNA methods developed herein represents an efficient and cost-effective way to audit their composition.

## Introduction

Traditional Chinese medicines (TCMs) have been an integral part of Chinese culture and the primary medicinal treatment for a large portion of the population for more than 3000 years [1], [2]. Outside of Asia there has been, in recent decades, a growing use of TCMs where they are being taken in conjunction with, or as an alternative to, conventional Western medicine [3], [4]. The increasing popularity of TCM products has seen the monetary value of the industry increase to hundreds of millions of dollars *per annum* [5], its growth paralleled by the global increase in the use of complementary and alternative medicines. Despite its increased uptake, the therapeutic benefits of only a small number of TCM products have been scientifically validated [6], with their perceived efficacy being based largely on long-standing beliefs [7].

Chinese herbal medicines often contain numerous different plant and animal-derived products that combine to act synergistically to affect a desired outcome [8], [9]. However, due to the proprietary nature of TCM manufacture, coupled with a lack of industry regulation, the biological origin of contents can be difficult to determine with confidence, leading to questions regarding TCM quality, efficacy and safety [10], [11]. Undeclared or misidentified TCM ingredients and adulterants can pose serious health risks to consumers [10], [12], [13]. These include: allergenic substances [14], plant toxins [7], heavy metals such as mercury, lead, copper and arsenic [15], and pharmaceutically active compounds of undetermined concentration [5]. In the early 1990s the misidentification of the toxic herb *Aristolochia fangchi* for the anti-inflammatory agent *Stephania tetrandra* led more than a hundred women to suffer kidney failure, with many later developing cancer of the urinary system [13].

In addition to safety concerns, issues of legality also surround TCMs. These concerns fall into three main categories: matters relating to the trade of endangered species; issues pertaining to honesty of food labelling; and adulteration of samples with drugs. Some TCMs contain plant and animal species [16]–[18] that fall under the jurisdiction of the Convention on International Trade in Endangered Species (CITES). CITES-listed species (see appendicies at www.cites.org) that have had long-standing associations and use within TCM include: Asiatic black bear (*Ursus thibetanus*, Appendix I listed), Saiga antelope (*Saiga tatarica*, Appendix II listed), rhinoceros (all species, Appendix I listed), and non-cultivated varieties of the plant genus *Panax*; *P. ginseng* and *P. quinquefolius*, (Appendix II listed) [19]–[23]. The CITES appendices include lists of species afforded different levels or types of protection from over-exploitation. Appendix I species are deemed the most endangered and threatened with extinction, with Appendix II and III listed species regarded to be at lower, but still significant, threat levels [24]. With an increased international demand for TCMs, ascertaining the biological origins, and hence the CITES status, of ingredients contained variously in capsules, powders, liquids, and tablets represents a complex problem for customs officials. The second issue of legality concerns the mislabelling of TCMs. This might be done intentionally in order to reduce manufacturing costs, or to circumvent customs' scrutiny, or inadvertently if the TCM practitioner unwittingly uses a misidentified product [25]. For CITES member states to enforce legislation and to prosecute cases of illegal trade, reliable methods of species identification are needed [26]. Lastly, a number of TCM products appear to have been intentionally adulterated with drugs of known pharmacological activity such as anti-hyperglycaemic agents (anti-diabetic medication) and corticosteroids [5], presumably as a means to increase their efficacy.

To date, many of the analyses and identification of botanical components in TCM products have employed chromatographic methods [9], [27]. However, these methods may not be able to identify animal species, or be able to uncover all of the ingredients within heterogeneous samples. DNA technology has the potential to provide information about species composition and the honesty of ingredient declarations. For the identification of botanical constituents used in TCMs, the genetic techniques employed include fragment length polymorphism analysis, dot-blot hybridization, micro-arrays, and sequencing of plastid DNA genes [25], [28]–[33]. Likewise, genetic identification of animal species commonly involves DNA sequencing and characterisation of mitochondrial DNA (mtDNA) genes [1], [32], [34]. Despite the variety of genetic work that has been conducted to date, investigative research seems to have focused on detecting the DNA of specific targets within TCMs [22], [28], [30], [35]–[38] or herbal teas [39] rather than investigating *all* of the contributing species within a sample simultaneously.

The advent of Second Generation, high-throughput sequencing (HTS) platforms have enabled the rapid sequencing of genes, genomes and metagenomes [40]. The ability of these technologies to deep-sequence both PCR amplified plastid and mtDNA markers (using molecular identifier [MID] tags) has allowed the species composition of a variety of complex substrates including faecal material [41], sediments [42] and even, in a forensic context, microbial communities on computer keyboards [43], to be determined. The application of HTS technologies to analyse complementary medicines has not been previously attempted, but is likely to prove to be the best approach by which to genetically audit the species composition of multiple TCM samples in parallel.

**Figure 1:**
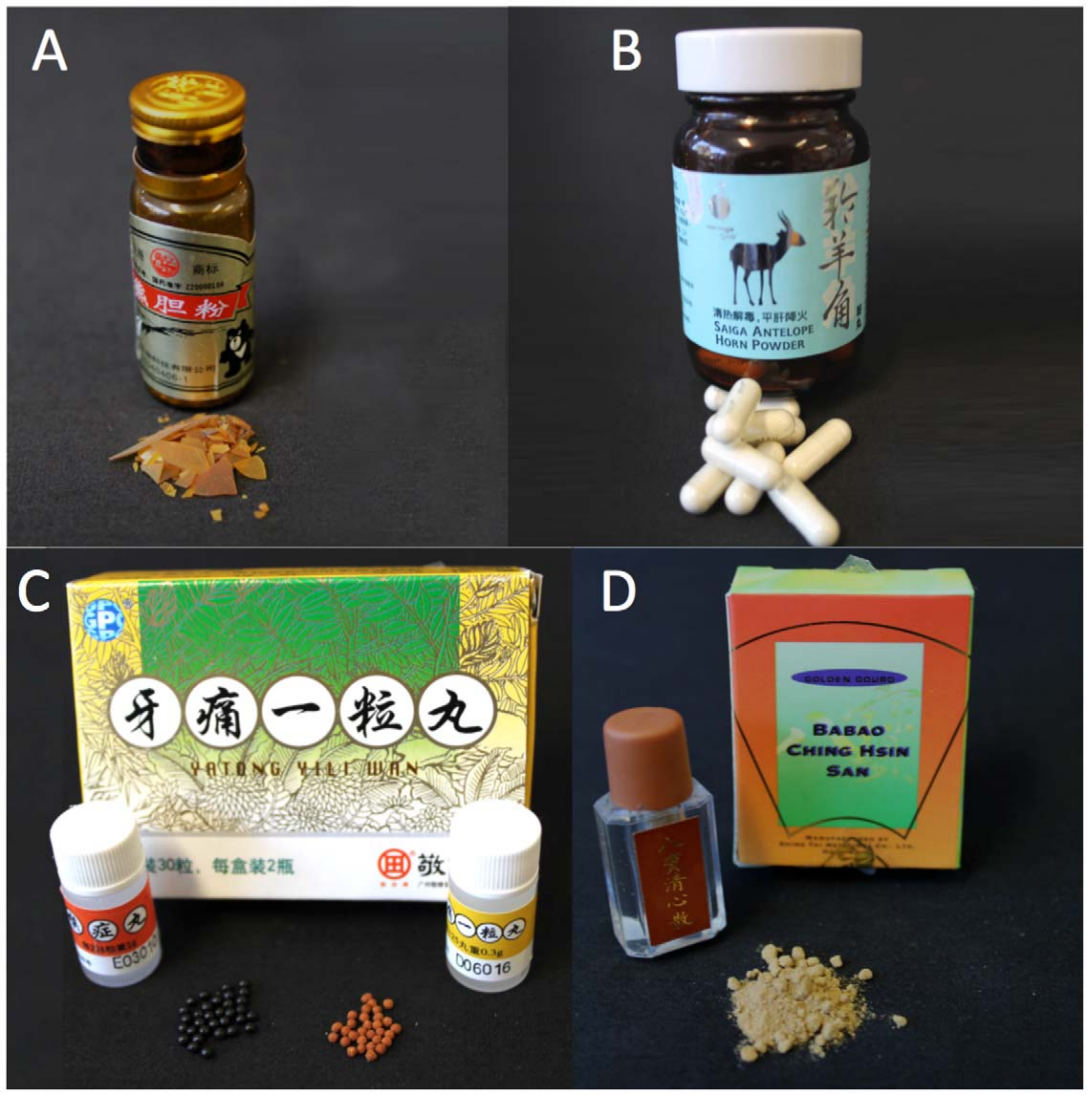
Photographs of four TCM samples genetically audited in this study using high-throughput sequencing. See Table 1 for a detailed list of all samples and listed package ingredients. From left to right; (A) Bear Bile crystals (TCM-015), (B) Saiga Antelope Horn powder (TCM-011), (C) Yatong Yili Wan capsules (TCM-016), and (D) Babao Ching Hsin San powder (TCM-026).

Given the worldwide popularity, growing use and increasing financial significance of TCMs, an effective means of evaluating these medicines is urgently needed – a sentiment echoed by strategy reports from the World Health Organization (WHO) [11]. This study sets out to explore the probative value of HTS approaches by generating species audits from 15 TCMs (Figure 1; Table 1) seized by border protection officials upon entry into Australia.

**Table 1:** Analysed TCM samples, including sample ID, brand names, and listed package ingredients.

Sample ID	Sample Name	Packet Ingredients (as they appear in English)	Packet Ingredients (Translated from Chinese)
TCM-001	Mongnan Tianbao Pills	*Radix ginseng, cornu cervi pantolrichum, Hippocampus, radix morindae officinalis, testis et penis callorhini, rhizoma curculinginis, herba cistanches, radix polygoni multiflori, semen cusculae, herba epimedii*	*Same as English listing*
TCM-002	Kai Yeung Pills	*Astragalus refiexistipulus, ligusticum acutilbum, agkistroban acutus, cnidicum officinale, panax ginseng, angelica anomala pall, atractylis lancas var. oveta, selinum japonicum miq, yanthium strumarium, phellodendron amurense, rehmannia glutinosa liboach, zaocys dhumnades, golden coin tortoise, moachus, agkistrodon, calculus bovis, squama manitis, fructus liquidamberis*	*Same as English listing*
TCM-003	Ling Yang Ge Gen Cold Remedy	*Fructus forsythiae, radix ledebouriellae, flos lonicerae, pericarpium citri reticulatae, radix platycodi, folium mori, herba menthae, folium perillae, radix glycyrrhizae, radix puerariae, herba schizonepetae, cornu antelopis, fructus arctii, fructus momordicae*	*Same as English listing*
TCM-004	Capsulae Bearbile	*No English translation for ingredients list*	*Rhizoma Coptidis powder, Rheum officinale (rhubarb), Chrysanthemum, Angelica sinensis, menthyl of brain*
TCM-006	Laryngitis Pills - A	*Borax, Coptis rhizome, Toad cake, Cow bezoar, Pearl, Musk, Bear gall*	*Same as English listing*
TCM-011	Saiga Antelope Horn Powder	*100% pure Saiga Antelope horn powder*	*Horns of wild animals: stage, antelope, rhinoceros*
TCM-013	Lingxin Mingmu Shangging Wan	*No English translation for ingredients list*	*Rhizoma Coptidis, Rheum officinale (rhubarb), China Clay/Plaster of Paris/Gypsum, Chrysanthemum, Herba Menthae (powder), Orange Peel, Angelica sinensis, Cicada*
TCM-015	Bear Bile Powder	*Bear bile powder*	*Same as English listing*
TCM-016	Yatong Yili Wan	*No English translation for ingredients list*	*Cicada oil/essence, pearl grains, liquorice, Synthetic Rheum officinale (rhubarb), pigs' bile, borax, cicada in wine*
TCM-018	Qingxuan Pian	*No English translation for ingredients list*	*Herba Menthae, China Clay/Plaster of Paris/Gypsum*
TCM-020	Zhen Zhu plus Hou Zao San Powder	*Radix siemona sessilifolia, fructus preilla frutescens, fructus amomi rotundus, radix aster tataricus, ramulus uncana cum uncis, cordyceps sinensis, pericarpium citri reticulata, bulbus fritillaria cirrosa, radix platycodon grandiflorum, calculus macaca*	*Only in English*
TCM-021	Laryngitis Pills - B	*Borax, Coptis rhizome, Toad cake, Cow bezoar, Pearl, Musk, Bear gall*	*Same as English listing*
TCM-024	Powder in 2 vials kept in red box with bear outline	*No English translation for ingredients list*	*Bear gall, goose bile, gall wine, gall powder*
TCM-026	Babao Ching Hsin San powder	*Pearl, bezoar, succinum, monkey bezoar, borneol, chamoishorn, cinnabaris, chuan pei, poria, scorpion, peppermint, rhynchophylla, radix bupleuri, herba ephedra, rhizoma atractylodis macrocephalae, rhizoma gastrodia, herba agastachis, rhizoma coptidis, radix platycodi, hairy kind of sage, radix sileris, jianghuo, agalloch, pinellia tuberifera, putchuck, jiangchai, radix scutellariae, periostracum cicadae, arisaema with bile, minerals, others*	*Same as English listing*
TCM-027	Chu pak hou tsao san powder	*Monkey bezoar, pearl, amber, bezoar, borneol, bulbus fritillariae cirrhosae, asarum sieboldi, carrdamomun, rhynchophylla, acorius granineus, gleditschia japonica, radix glycyrrhizae, citrus nobilis lour*	*Same as English listing*

The descriptions listed in the table are taken *verbatim*, from the respective TCM. The taxonomy, scientific nomenclature, and format are also exactly as listed, including any inaccuracies.

## Results/Discussion

### General overview of HTS results

An in-depth genetic audit of the species constituents contained within 15 TCM samples (Figure 1, Table 1) was determined by using amplification of *trnL* (p-loop, plastid) and 16S rRNA (mtDNA) genes, followed by deep sequencing via HTS (see methods). More than 49,000 sequence reads were obtained from the HTS approach using both *trnL* c/h and 16S primers, with the analysis of the plant and animal constituents discussed separately below. The DNA isolated from the various TCM samples was highly variable in quality. Using *trnL* and 16S primers in qPCR assays, DNA of sufficient quality was obtained from 15 of 28 (54%) samples attempted. Some of the TCMs failed to amplify due to severe PCR inhibition, while others yielded little, or no DNA. As with many other degraded/processed substrates it may be necessary to optimise DNA extraction methodologies depending on the physical and chemical properties of the TCM.

To our knowledge, this is the first study to apply an HTS approach to ascertain the species composition of medicinal products. Until recently, to dissect the molecular components of heterogeneous biological samples (such as TCMs) it has been necessary to clone amplicons into plasmid vectors and then sequence the insert. In direct contrast to previous cloning based methodologies HTS provides deeper coverage of more samples in a shorter time period, and represents a cost effective way to audit DNA in heterogeneous samples. The sequencing of indexed (MID-tagged) PCR amplicons [44] allows for the sequencing of multiple samples in parallel, with the GS Junior or Ion Torrent conservatively generating ∼50,000 reads for *c.* US$1000 [45]. DNA isolation and quantification of 15 TCM samples followed by a single HTS run of the pooled and tagged PCR products, was estimated, in this case, to cost less than $35 per sample (excluding labour). This demonstrates that after an initial outlay for MID-tagged primers this approach is extremely cost-effective. As such, the approach described here is both cost-effective, accessible, and can be easily adapted to profile the molecular constituents of other biologically derived complementary and alternative medicines. One of the aims of this study was to determine the efficacy of HTS auditing approaches specifically with the goal of screening additional samples whose constituents might need to be identified in cases involving illegal imports, food fraud, medicine fraud and forensics.

Taxonomic assignment of DNA sequences to a family, genus or species represents a complex problem, the accuracy of which largely depends on the level of coverage afforded by reference databases, the analytic method used [46] and the accuracy of the underlying taxonomic framework. In the TCM data generated here the vertebrate assignments were relatively straight forward, in contrast to the plant assignments, which were particularly challenging. The detection and identification to the family level, of genetically well-characterised plants and animals is generally uncomplicated. In contrast, if species-level assignments (without uncertainties) are required for each *trnL* sequence, the task is largely unachievable with current databases. While the MEtaGenome ANalyzer (MEGAN) [47] based assignment approach is not without problems, it is currently the best way to parse thousands of sequence reads. Alternative methods for assigning sequences are also available such as SAP [48] and QIIME [49] although all of these methods are computationally intensive when challenged with large volumes of data. Irrespective of the species assignment methodology used, whether it be phenetic or character-based, all are ultimately dependent on good reference database coverage and a robust taxonomy.

There are a number of caveats with regards to HTS technology that need to be considered when analysing data. Firstly the error rate of 454 Titanium chemistry is estimated to be ∼0.5–1% [50]. On top of this there is the possibility that recombination might occur, albeit at a low (∼0.3% on an Illumina platform) frequency [51]. The likelihood of error and recombination should at least be acknowledged, but with respect to the plastid *trnL* data presented here it is debatable how significant an impact this is going to have on species assignments due to the presence of both sequence and length polymorphisms in the p-loop region. Lastly, caution also needs to be exercised with drawing correlations between the genetic profiles detected by HTS approaches and the *actual* composition of the TCM. No genetic audit can detect DNA when it has been completely degraded (for example by processing procedures) and there will always be variation in the DNA concentrations between ingredients. The results should therefore be regarded as a qualitative, and potentially incomplete assessment of composition rather than a quantitative measure of each ingredient.

Within the confines of a manuscript it is impossible to document the significance of each of the ∼50,000 reads in this audit, instead, a summary of the data is presented (Table 2 and Table 3, and Figure S1A–S1N) and the discussion will focus on some of the more common, illegal or hazardous ingredients.

**Table 2:** Selected plant families and genera identified in 13 TCM samples using HTS.

Sample ID	TCM-001	TCM-002	TCM-003	TCM-004	TCM-006	TCM-011	TCM-013	TCM-016	TCM-018	TCM-020	TCM-021	TCM-026	TCM-027
Number of DNA sequences	7444	563	3964	4944	7619	4445	4421	2123	2112	850	7454	912	1831
**Anacardiaceae**					✓						✓		
**Apiaceae**	✓	✓	✓			✓			✓			✓	
- *Cymopterus nivalis*									✓*				
**Araceae**						✓		✓					
**Araliaceae**	✓					✓			✓				✓
**Aristolochiaceae**					✓		✓	✓			✓		
- *Asarum*					✓*		✓*	✓*			✓*		
**Asteraceae**		✓	✓		✓	✓	✓	✓		✓	✓		
**Brassicaceae**		✓				✓		✓			✓		
*- Isatis*								✓*					
**Caprifoliaceae**													✓
- *Lonicera*													✓*
**Cucurbitaceae**			✓					✓	✓	✓			
**Cyratophyllaceae**								✓					
*- Ceratophyllum demersum*						✓*		✓*					
**Elaeagnaceae**								✓					
**-** *Hippophae tibetana*								✓*					
**Ephedraceae**								✓					
- *Ephedra*								✓*					
**Fabaceae**		✓	✓		✓	✓	✓	✓	✓	✓	✓	✓	
**-** *Glycyrrhiza uralensis*		✓*	✓*		✓*	✓*	✓*	✓*		✓*		✓*	
*- Glycine max*						✓*	✓*	✓*			✓*		
*- Sophora flavescens*						✓*							
*- Vigna*								✓*					
**Lamiaceae**		✓	✓		✓	✓	✓	✓		✓	✓	✓	✓
**-** *Mentha*			✓*			✓*	✓*			✓*		✓*	✓*
**Moraceae**			✓										
*- Morus alba*			✓*										
**Oleaceae**			✓			✓							
*- Jasminum*						✓*							
**Paeoniaceae**							✓						
- *Paeonia*							✓*						
**Poaceae**			✓		✓	✓	✓	✓		✓	✓	✓	✓
*- Sorgum bicolor*							✓*						
*- Hordeum*													✓*
**Polygonaceae**		✓		✓	✓								
**Ranunculaceae**				✓	✓		✓		✓		✓		
*- Xanthorhiza simplicissima*					✓*		✓*						
*- Epimedium myrianthum*									✓*				
**Rosaceae**			✓		✓	✓		✓			✓		
*- Agrimonia eupatoria*						✓*							
*- Prunus*								✓*					
**Rutaceae**	✓						✓	✓					
*- Zanthoxylum*	✓*												
**Scrophulariaceae**			✓			✓							
*- Manuleae*			✓*			✓*							
**Solanaceae**			✓		✓	✓			✓				
**Zingiberaceae**	✓												✓
- *Plagiostachys mucida*													✓*

*: indicates top BLAST match ≥98%. A comprehensive audit is shown in Figure S1A–S1N.

**Table 3:** Animal genera identified in the TCM samples using HTS.

Sample ID	Number of DNA sequences (excluding *Homo*)	*Ursus thibetanus* (Bear) #	*Saiga tatarica* (Saiga antelope) #	*Bufo* (Asiatic Toad)	*Capra* (Goat)	*Ovis* (Sheep)	*Cervus elephas* (Deer)	*Bubalus* (Buffalo)	*Bos taurus* (Cow)
TCM-001	48						✓*		✓*
TCM-003	59				✓*	✓*			
TCM-006	124			✓*				✓	
TCM-011	24		✓*		✓*	✓*			
TCM-015	29	✓*							
TCM-016	32			✓*					
TCM-021	52			✓*				✓*	
TCM-024	98	✓*							
TCM-027	73	✓*							

*: indicates top BLAST match ≥98%. The symbol # denotes that the species are CITES listed [24].

### Analysis of plant DNA in the TCM samples

A total of 68 plant families were identified in this study with 48,682 DNA sequence reads (on average 3,745 per TCM sample) generated using the *trnL* c/h primer set [52] for the 13 analysed samples (Table 2). Six of the most common plant families that were identified included Fabaceae, Asteraceae, Poaceae, Lamiaceae, Solanaceae, and Apiaceae, with 70% of the samples containing at least three of these families (Table 2). Some of the most common plant genera identified in the TCM samples were *Glycyrrhiza* (liquorice root, Family Fabaceae), found in 62% of samples, *Mentha* (mint, Family Lamiaceae), found in 46% of samples and *Asarum* (wild ginger, Family Aristolochiaceae) found in 31% of samples. Mint is commonly included in medicines and is used in TCM to treat gastrointestinal upset, gallbladder problems and upper respiratory symptoms [53]. Likewise *Glycyrrhiza uralensis*, or Chinese liquorice root, is a common component of TCM remedies and is classified as one of the Chinese 50 fundamental herbs [54]. Containing glycyrrhizin, *G. uralensis* can be processed by microbes into 18β-glycyrrhetic acid — effective in the treatment of peptic ulcers, as well as having antiviral and antifungal activities [55]. Heavy harvesting of *G. uralensis* from the wild for TCM products, has resulted in the threat of species extirpation in Chinese provinces such as Gansu [56].

**Figure 2:**
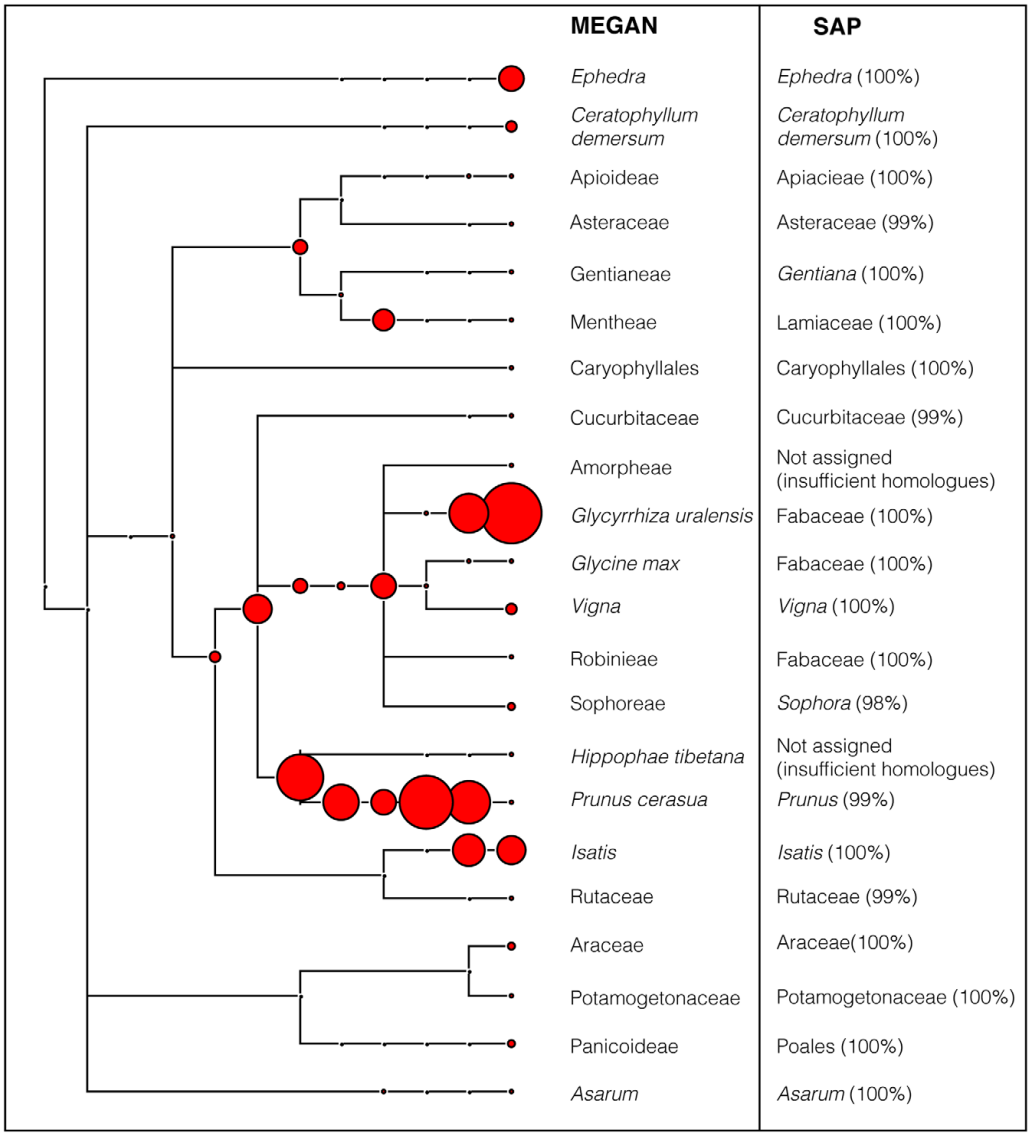
MEGAN phylogram of plant components in Yatong Yili Wan capsules (TCM-016). The data was generated using *trnL* c/h fusion primers and HTS using the Roche GS Junior. 2123 reads were queried against GenBank and parsed through MEGAN, SAP and QIIME (see Methods). The assignments of both MEGAN and SAP (with posterior support) are shown. Size of red node labels is proportional to number of sequence reads at each taxonomic level.

The results of the *trnL* audit on four samples, Yatong Yili Wan (TCM-016), Laryngitis pills (TCM-006, TCM-021), and Lingxin Mingmu Shangging Wan (TCM-013), indicated they contained DNA with close (>98%) similarity to the genera *Ephedra* and/or *Asarum* (Table 2). These TCMs could potentially pose a risk, as compounds from these genera can be poisonous or toxic at high dosages. *Ephedra* is classed as a poisonous herb, with *Ephedra*-containing products having been banned by the U.S. Food and Drug Administration (FDA) since 2004 [57]. Remedies that contain *Ephedra* should only be prescribed by experienced practitioners, as the therapeutic dose range is narrow [8]. Aristolochic acid, the same compound as contained in *Aristolochia* species, a known nephrotoxin, hepatotoxin, and carcinogen [27], [58], may be contained in certain species of *Asarum*. Further compound specific testing (via metabolomics) of TCMs from which *Asarum* DNA was detected (TCM-006; TCM-013; TCM-016; TCM-021, Figure 2, Table 2) would be required to determine whether this acid is actually present in the TCMs analysed here.

One trade-restricted plant species commonly found in TCM preparation is *Panax ginseng* (CITES Appendix II). Non-cultivated *P. ginseng* is subject to CITES regulation only when in the form of a whole root, or sliced parts of the root, and not after processing and manufacture [23]. It was not possible using the conservative assignment criteria implemented in MEGAN to definitively identify the genus *Panax*, this is primarily because the bit-score match was equally good with the genus *Hedera* (ivies). Both *Panax* and *Hedera* are in the family Araliaceae and further molecular characterisation is required to distinguish if one or both of these genera are present in the TCM-001, TCM-011, TCM-018 and TCM-027. Even if *Panax* is confirmed, the fact that all the TCMs containing Araliaceae sequences are in powdered form render them technically not subject to CITES legislation.

Additional plant taxa with purported medicinal activity identified in the samples include *Xanthorhiza simplicissima* (Ranunculeae), and *Sophora flavescens* (Fabaceae). *Xanthorhiza simplicissima* (Yellowroot) is a native American medicinal containing berberine which is anti-inflammatory, astringent, hemostatic, antimicrobial, anticonvulsant, immunostimulant, uterotonic and can temporarily lower blood pressure [59]: the roots of *Sophora flavescens* contain alkaloids such as oxymatrine and is commonly used to treat fever, asthma, cancer and viral myocarditis [60], [61]. Plant DNA assigning to the families Cannabaceae, Ranunculaceae, and Solanacea, which are known to contain medicinally important species, were also recovered. However to resolve these sequences beyond the family level another gene region would need to be targeted, and this might reveal, for example, whether the Solanaceae (Nightshade family) identified in four of the TCM samples comprised *S. chrysotrichum* (Giant Devil's Fig) which has known pharmacological activity [62], or perhaps the less exotic species such as potato or tomato.

The complexity and risk of possible drug interactions for consumers using TCMs in combination with conventional medicines could be heightened when there are poisonous or toxic ingredients of unknown concentrations in herbal remedies that may not be listed on the packaging (Table 1). Further to potential adverse drug interactions is the possibility of ingesting allergenic substances within herbal remedies, such as nuts, which can cause anaphylaxis in those with severe allergy. DNA from the Anacardiaceae (the cashew or sumac family) was detected in two TCMs - nut proteins from this family are know allergens [63]. Likewise, *Glycine* (soybean) was detected in four TCMs and is known to contain at least 16 potential protein allergens with the potential to cause adverse reactions ranging from mild rashes to life threatening systemic anaphylaxis [64]. However, our results were unable to determine whether the recovered DNA is derived directly from the nut/bean, or originates from other plant tissue.

The variety of species that the HTS technique can reveal when analysing TCMs, is demonstrated by the results obtained for the Yatong Yili Wan pills (TCM-016). This sample was one of the most botanically complex, containing 16 identifiable plant families. 2,124 DNA sequence reads, were assigned to a GenBank reference database sequence (Table 2; Figure 2), based on cut-offs in MEGAN (see methods). SAP analysis was also conducted on representative sequences from each of the terminal nodes. Results generated by SAP were in close accordance with the MEGAN assignments with high posterior support. The two cases where no assignment was made was the result of insufficient database coverage – the method is reliant upon sufficient sequence coverage to construct a phylogeny. A third assignment method was also implemented, QIIME, the results of which were also in broad agreement with the MEGAN and SAP assignments (Figure 2).

What is clear from the plant assignments of the HTS data is that better reference databases involving multiple genes (such as: *trnL*, *rbc*L, ITS and *mat*K) are required to improve the species assignment. A medicinal materials DNA barcode database (MMDBD) is currently being generated and compiled to include thousands of DNA reference sequences for these and other genes covering species of plants, animals, insects and fungi that are commonly used in TCM (available at; http://www.cuhk.edu.hk/icm/mmdbd.htm) [31]. The recent work of the China barcode of life group [65] which has sequenced ∼6000 species may soon rectify inadequacies in the plant databases. Despite the constantly improving databases, the taxonomic framework under which the DNA assignments operate also needs to be scrutinised. What is reassuring about HTS data is that while the resolution may not currently be available, efforts to improve databases and the underpinning taxonomies are continually improving and hence the accuracy of assignments can only get better.

With the potentially enormous volumes of plant data produced (over 7,662 reads in the case of TCM-006), it is tempting to look for quantitative signals in results, but owing to various factors including differential preservation of DNA in the raw ingredients, different processing techniques, variation in PCR efficiency (due to amplicon length variation and primer binding site polymorphisms), a universal primer approach should be viewed as semi-quantitative at best. In the worst-case scenario a constituent may be entirely undetected, especially if it occurs at a very low abundance.

### Analysis of vertebrate DNA in the TCM samples

With the exception of human-derived sequences (which were excluded), vertebrate genetic signatures were detected in nine samples tested using two universal 16S rRNA primer pairs [66], [67]. A total of eight animal genera were identified from 539 DNA sequences (Table 3). The taxonomic assignments of the vertebrate sequences were simpler in comparison to the plant assignments, due to substantially better GenBank coverage, but as with other forensic studies caution still needs to be exercised when assigning a species in casework [68], [69]. This study identified four TCM samples - Saiga Antelope Horn powder (TCM-011), Bear Bile powder (TCM-015), powder in box with bear outline (TCM-024) and Chu Pak Hou Tsao San powder (TCM-027) – that were found to contain DNA from known CITES listed species. Two of these CITES species are classified by the IUCN Red List as vulnerable (*Ursus thibetanus*) and one as critically endangered (*Saiga tatarica*) (Table 3). The threat posed to these and other animal species' survival caused by the demand for TCM products is high [7], [18]. This highlights a serious concern for the conservation of these species and it is evident that illegal hunting still persists despite a high level of legal protection [70]. One hundred and seventy five countries are signatories to CITES, including China (member party since 1981) [24], yet penalties for illegal trafficking are relatively minor and penalties are rarely enforced [18]. DNA testing of highly processed medicines may assist in the successful prosecution of individuals who seek to profit from the illegal trade in endangered taxa. Likewise, such genetic screens will help to legitimise those medicines that contain components that are not trade restricted, but may still be confiscated on grounds of suspicion (e.g. TCM-003, 006 and 021).

Of the samples analysed using the 16S rRNA primers, 44% contained two or more animal species within the same sample (Table 3). Some of these species, such as water buffalo (*Bubalus bubalis*), Asiatic toad (of the genus *Bufo*), and domestic cow (*Bos taurus*), are known for their use in medicinal products [27], [71], whereas use of goat (*Capra hircus*) is less well represented in the literature and may be used as a substitute for traditionally used animal species. As with all animal-containing products the consumer needs to be aware of the possibility of zoonotic pathogens, such concerns have been raised previously in the context of TCM [7].

Consumers of TCMs need to be wary of honesty of food labelling [72], as in 78% of samples, animal DNA was identified that had not been clearly labelled on the packaging (in either English or Chinese). This adulteration of medicine occurred in the Saiga Antelope Horn powder (TCM-011; Table 1) which claimed to be 100% pure, yet was found to also contain significant quantities of goat (Caprine) and sheep (Ovine) DNA (Table 3). In some TCMs, undeclared ingredients are used to reduce the cost of manufacture of the medicine by increasing the bulk of the powder, but it is impossible to determine why Caprine and Ovine appeared in this product. Water buffalo (*Bubalus bubalis*), domestic cow (*Bos taurus*) and deer species were also not listed on the packaging of samples in which they were genetically identified (Table 1 and 3). The inadvertent consumption of undeclared animal products found in 78% of the medicines, such as bovid, risk violating certain religious and/or cultural strictures.

### Concluding remarks

The results of this study demonstrate that high-throughput DNA sequencing methods are an invaluable tool for analysing constituents within complex TCMs. The techniques used enabled the identification of a larger number of animal and plant taxa than would have been possible through morphological and/or biochemical means. HTS methodology is well suited to the analysis of highly processed and degraded DNA from TCMs, including powders, crystals, capsules, tablets, and herbal tea. It is manifestly obvious that if there are trade-restricted biological materials in TCMs, or if they contain DNA from species known to synthesise toxic compounds, that better methods of detection are urgently required. Even in the 15 TCMs tested here, the occurrence of CITES-listed species, potentially toxic/allergenic plants and non-declared constituents was all too common. However, it should also be noted that the detection of DNA from a pharmaceutically active species does not necessarily indicate the presence of bioactive compounds: metabolomic analyses can be used in addition for the detection of specific compounds. For example, the bear-bile powder (TCM-015; Table 1 and Table 3) containing Asiatic black bear DNA was analysed using Gas Chromatography Mass Spectrometry and yielded a mass spectra consistent with ursodeoxycholic acid (data not shown), an active component of bile that has been reported to reduce pain and inflammation [73].

In the future, TCM screening approaches that involve *both* genetic (for species composition) and metabolomic (for compound detection) approaches could represent the best way to audit complementary medicines. With regard to TCMs and complementary medicines as a whole, controls need to be implemented to ensure consumer safety and to minimise impacts on protected biota. It is also important that consumers are made fully aware of legal and health safety concerns that surround TCMs before adopting them as a treatment option. A recent opinion piece [74] stated “*if TCM is to take its place in the modern medicine cabinet, then it must develop ways to prove itself*” – we endorse this view and note that it applies equally to safety as it does to medical efficacy.

## Materials and Methods

### Sample collection, DNA extraction, and quantification

Twenty-eight TCM samples were obtained from the Wildlife trade section of the Department of Sustainability, Environment, Water, Population and Communities after being seized by Australian Customs and Border Protection Service at airports and seaports across Australia. The samples were seized because they contravened Australia's international wildlife trade laws as outlined under Part 13A of the Environment Protection and Biodiversity Conservation Act 1999 (EPBC Act). The samples were stored in a quarantine-approved facility within the laboratory after being catalogued. TCM sample types included: powders, bile flakes, capsules, tablets, and herbal tea. Small amounts of each sample (between 70–290 mg) were dispensed into 2.0 mL Eppendorf tubes and digested overnight, on a shaking heat block at 55°C, in 700 µl–1500 µl of tissue digest buffer consisting of; 1 mg per mL proteinase K (Amresco, OH, USA), 20 mM Tris pH 8.0 (Sigma, MO, USA), 2.5 mM EDTA (Invitrogen, CA, USA), 5 mM CaCl_2_ (Sigma), 20 mM DTT solution (Thermo Fisher Scientific, MA, USA), 1% SDS (Invitrogen), and milliQ water.

All samples were centrifuged after digestion for 3 minutes at 16,813×g. 200 µL of supernatant was mixed with 1 mL of Qiagen (CA, USA) PB buffer and transferred to a Qiagen (PCR cleanup) spin column and centrifuged for 1 minute at 16,813×g. Two wash steps followed (Qiagen AWI then AWII buffer) prior to elution of DNA from the spin column membrane with 50 µL of 10 mM Tris pH 8.0. The DNA extracts were then quantified via real-time quantitative polymerase chain reaction (qPCR; Applied Biosystems [ABI], USA) using *trnL* g/h [52] and 16S ribosomal RNA (rRNA) [66], [67] primers (Integrated DNA Technologies [IDT], USA) (Primer sequences displayed in Table S1). Samples were assessed for quality and quantity of DNA using qPCR at three DNA dilutions (undiluted, 1/10, 1/100) to determine if successful isolation of DNA was achieved, and to investigate the presence of PCR inhibition. The *trnL* g/h qPCR assay was conducted in 25 µL reactions using ABI Power SYBR master mix together with 0.8 µM of *trnL* g and *trnL* h primers and cycled at 95°C for 5 minutes followed by 40 cycles of 95°C for 30 s, 50°C for 30 s, 72°C for 30 s, with a 1°C melt curve stage and a 10 minute final extension at 72°C. The 16S qPCR was conducted using the same conditions, except for the primer concentration used, which was 0.4 µM and an annealing temperature of 57°C. An optimal DNA concentration, free of inhibition was selected and used for further analysis. Samples with low template amounts and/or severe inhibition were not processed further.

### Amplicon generation

Fusion primers with unique 6 bp MID tags were designed [74] for both the 16S rRNA [65], [66] (∼150 bp product for 16Smam, ∼250 bp product for 16S1/2 degenerate primers [Table S1]) and the p-loop region of *trnL* [52] (c/h primers generating a size variable product averaging ∼250 bp product [Table S1]) (IDT, Australia). The *trnL* c/h primer sets were used to generate a longer PCR amplicon for future HTS, instead of the *trnL* g/h primer set (∼100 bp) which were only used for initial quantification. For the most part, when we used qPCR on the c/h and g/h primers, there were no significant drops in detected copy number. For this reason we selected the longer c/h set as it affords greater taxonomic resolution. Ten samples were PCR amplified using both the *trnL* c/h and 16S fusion primers, three samples were PCR amplified using *trnL* c/h fusion primers only, and two samples were PCR amplified with 16S fusion primers only. Amplicons were generated via PCR for each sample in triplicate (Corbett Research, NSW, Australia) and pooled in an attempt to reduce the effect of PCR stochasticity. The *trnL* c/h PCR was carried out in a 25 µL total volume including 4 µL of template DNA, with the following reagents: 2 mM MgCl_2_ (Fisher Biotec, Aus), 1× Taq polymerase buffer (Fisher Biotec, Australia), 0.4 µM dNTPs (Astral Scientific, Australia), 0.1 mg BSA (Fisher Biotec, Australia), 0.4 µM of each primer, and 0.25 µL of Taq DNA polymerase (Fisher Biotec, Australia). The PCR conditions were as follows: initial denaturation at 95°C for 5 minutes, followed by 50 cycles of 95°C for 30 s, 50°C for 30 s, 72°C for 30 s, and a final extension at 72°C for 10 minutes (Corbett Research, NSW, Aus). The 16S PCR was carried out in 25 µL total volume including 4 µL of template DNA, and the same dNTP, primer and buffer concentrations, but with 2.5 mM MgCl_2_, 0.4 mg BSA, and 0.25 µL of AmpliTaq Gold DNA polymerase (ABI) instead. The PCR conditions included: initial denaturation at 95°C for 5 minutes, followed by 40 cycles of 95°C for 30 s, 54°C 30 s, 72°C for 30 s, and a final extension at 72°C for 10 minutes (Corbett Research, NSW, Aus).

All PCR amplicons were double purified using the Agencourt AMPure XP Bead PCR Purification protocol (Beckman Coulter Genomics, MA, USA). The purified PCR amplicons were then electrophoresed together on the same 2% agarose gel to confirm the presence of the amplicons and to allow estimates of DNA concentration to be made based on comparisons between band intensity, prior to approximate equimolar amplicon pooling for emulsion PCR.

### GS Junior run set up for HTS

To achieve the desired bead∶template ratio, pooled PCR amplicons were quantified using a synthetic 200 bp oligonucleotide standard (of known molarity) with the Roche A and B primers engineered at either end [75]. Quantitative PCR on both the standard and the pooled library was required to approximate the optimal bead∶template ratio. The Roche GS Junior run set up included an emulsion PCR step, bead recovery, and the sequencing run. All of these procedures were carried out according to the Roche GS Junior protocols (http://www.454.com).

### Analysis of GS Junior HTS data

The sequencing output Fasta (.fna) and Quality (.qual) formatted files were processed using the following applications. Reads were quality trimmed using BARTAB [76] with a minimum acceptable quality score of 20, averaged over a window size of five bases, then separated into sample batches using a map file containing sample and primer-MID tag information. A non-redundant data set was also generated for each sample. The batched sample read primer and MID tag sequences were masked with the cross_match application [77], for minimum match length of 12 and minimum score of 20, then trimmed using trimseq [78]. An alternative means of data sorting was also employed and involved using the “separate by barcode” and primer trim feature in Geneious (v5.5). Once deconvoluted (based on MID tags) each batch of reads was searched using BLASTn version 2.2.23 [79] with a gap penalties existence of five and extension of two. The low complexity filter option was set to false, and the number of hits was limited to 100 and an expected alignment value less than 1e-10. The BLASTn search was against the National Centre for Biotechnology Information (NCBI) GenBank nucleotide NR database [80], containing all GenBank, EMBL, DDBJ and PDB sequences, a total number of 13,504,325 database sequence entries. This dataset contained no EST, STS, GSS, environmental samples or phase 0, 1 or 2 HTGS sequences, database posted date was Oct 6, 2010 5:44 PM. This pipeline was automated in an Internet-based bioinformatics workflow environment, YABI (https://ccg.murdoch.edu.au/yabi/). The resultant BLAST files were imported into the program MEtaGenome ANalyzer (MEGAN version 4.62.1) [47] for taxonomic analysis and assignment of amplicon plant and animal sequence data, using the following lowest common ancestor parameters: min score of 65, top percent of 5, and min support of 1. To compare the MEGAN assignments with other algorithms we conducted a SAP analysis [48] on a subset of data from TCM-016 where Bayesian trees were constructed from an alignment of at least 30 homologous sequences. QIIME [49] analysis was also implemented. However establishing a valid reference alignment file proved difficult for the *trnL* of some of the TCM taxa.

Data described herein is available in a processed and annotated form from Dryad Digital Repository: http://dx.doi.org/10.5061/dryad.8ps58rp2. Alternatively in its raw form from the short read archive – accession number SRA047476.

## Supporting Information

Figure S1 (A–N) MEGAN phylograms of plants identified in 13 TCMs after HTS of *trnL* c/h gene. The data parsed through MEGAN is illustrated at the lowest taxonomic level according to the LCA parameters used (see Methods). A summary figure which combines the BLAST results from all 13 TCMs also shown in (N). Data used to generate the phylograms can be obtained in a processed form from Dryad Digital Repository: http://dx.doi.org/10.5061/dryad.8ps58rp2.
(PDF)

Table S1 Mitochondrial and plastid primer sequences used in this study.
(PDF)
