# Supplementary figures and images for: Deep Sequencing of Plant and Animal DNA Contained within Traditional Chinese Medicines Reveals Legality Issues and Health Safety Concerns
Source: PLoS Genet. 2012 Apr 12;8(4):e1002657. doi: 10.1371/journal.pgen.1002657 (PMC3325194; doi:10.1371/journal.pgen.1002657)

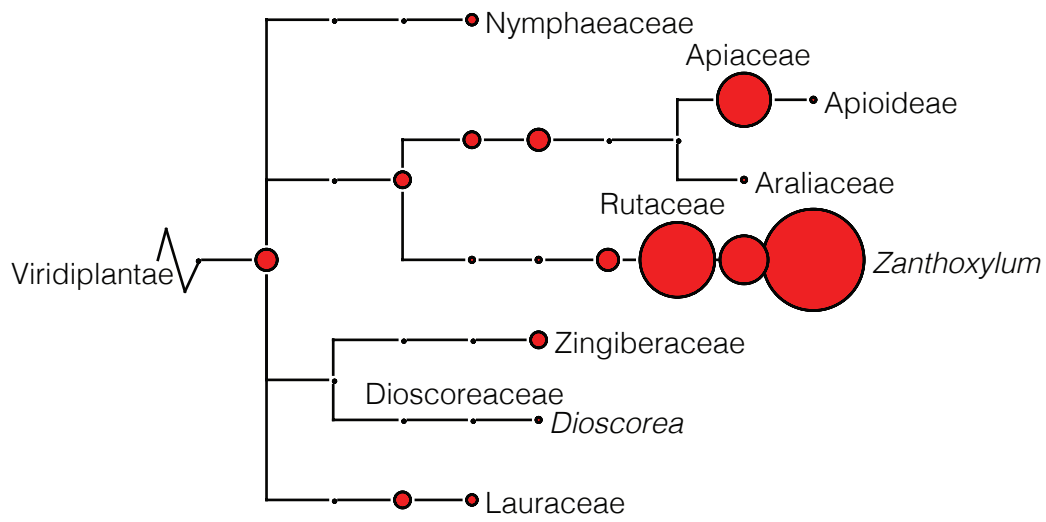

**A. TCM-001**

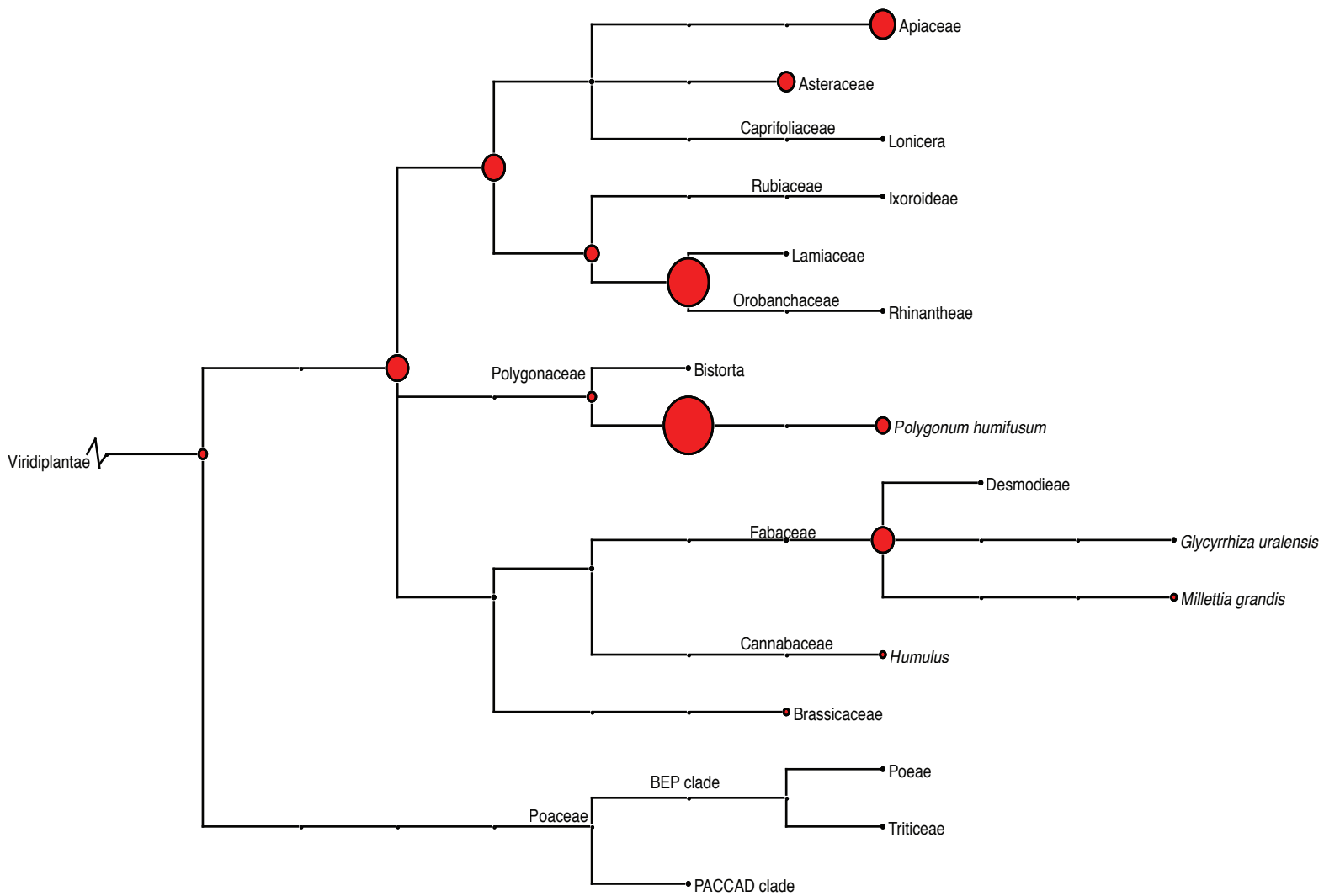

**B. TCM-002**

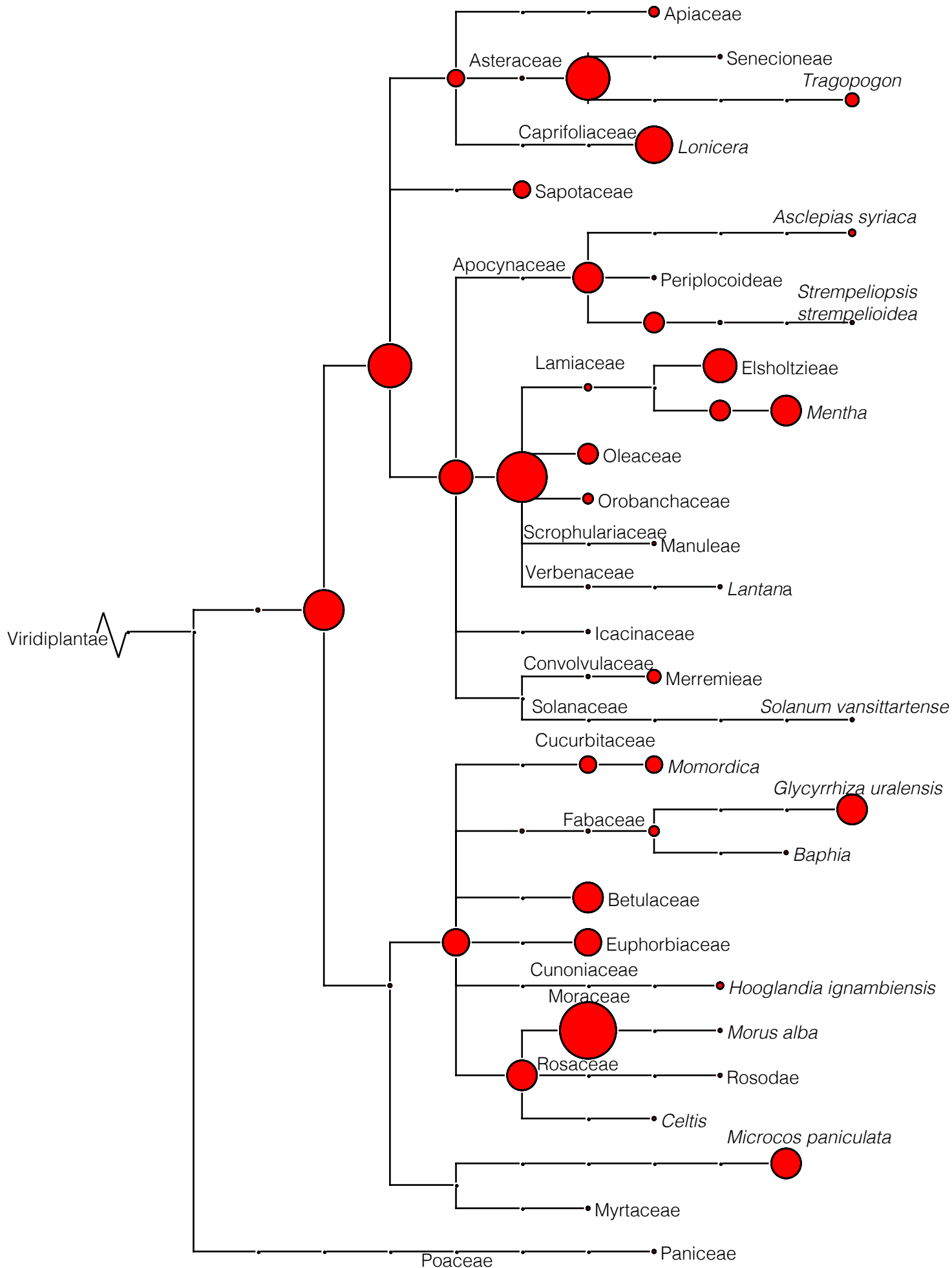

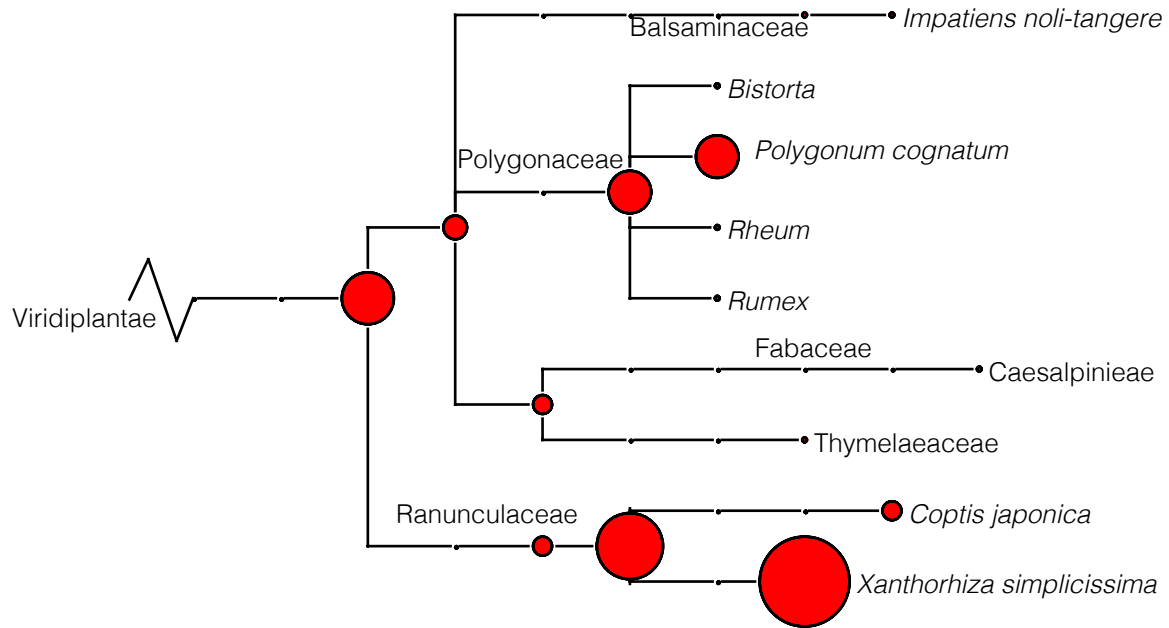

#### D. TCM-004

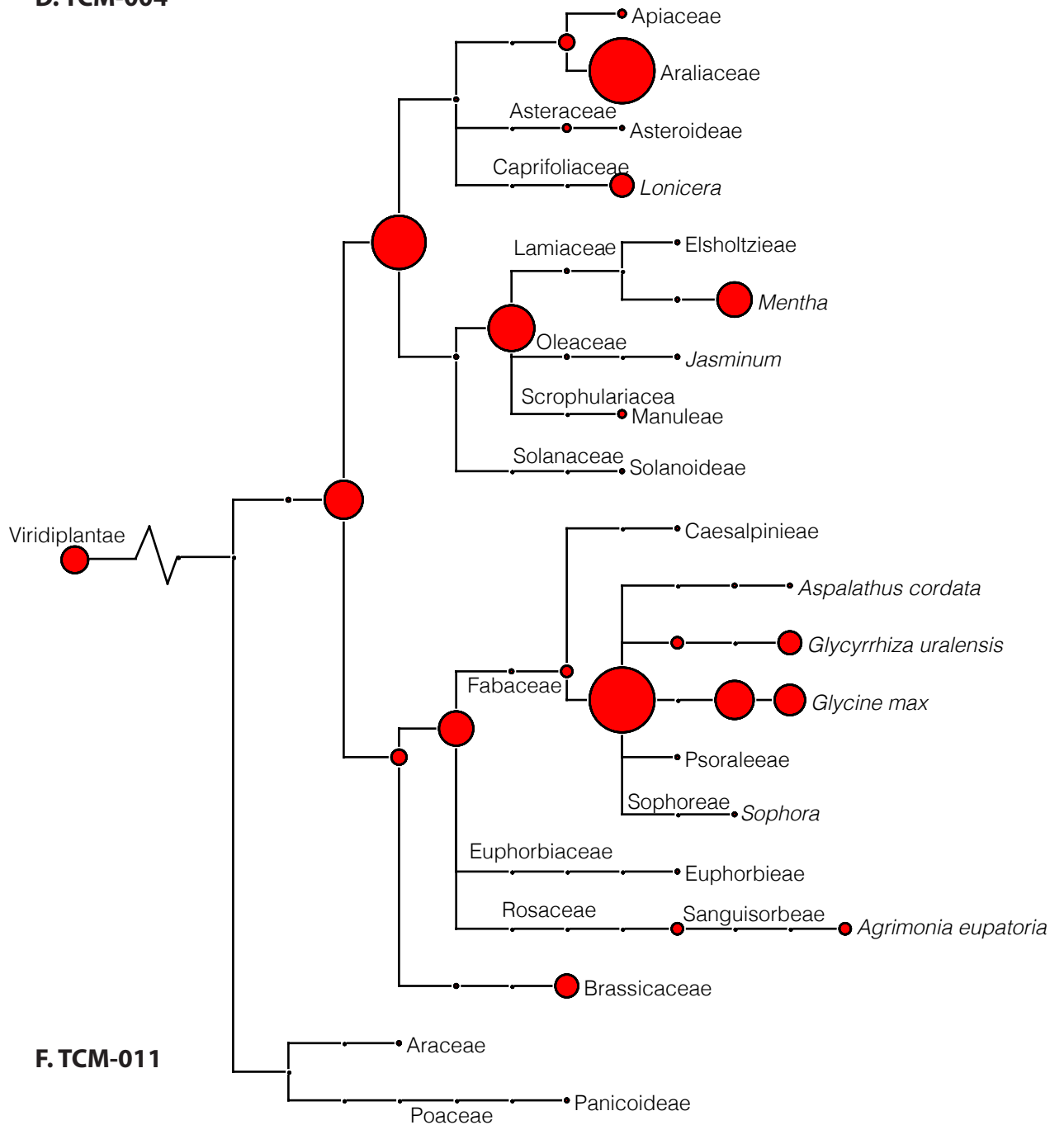

#### F. TCM-011

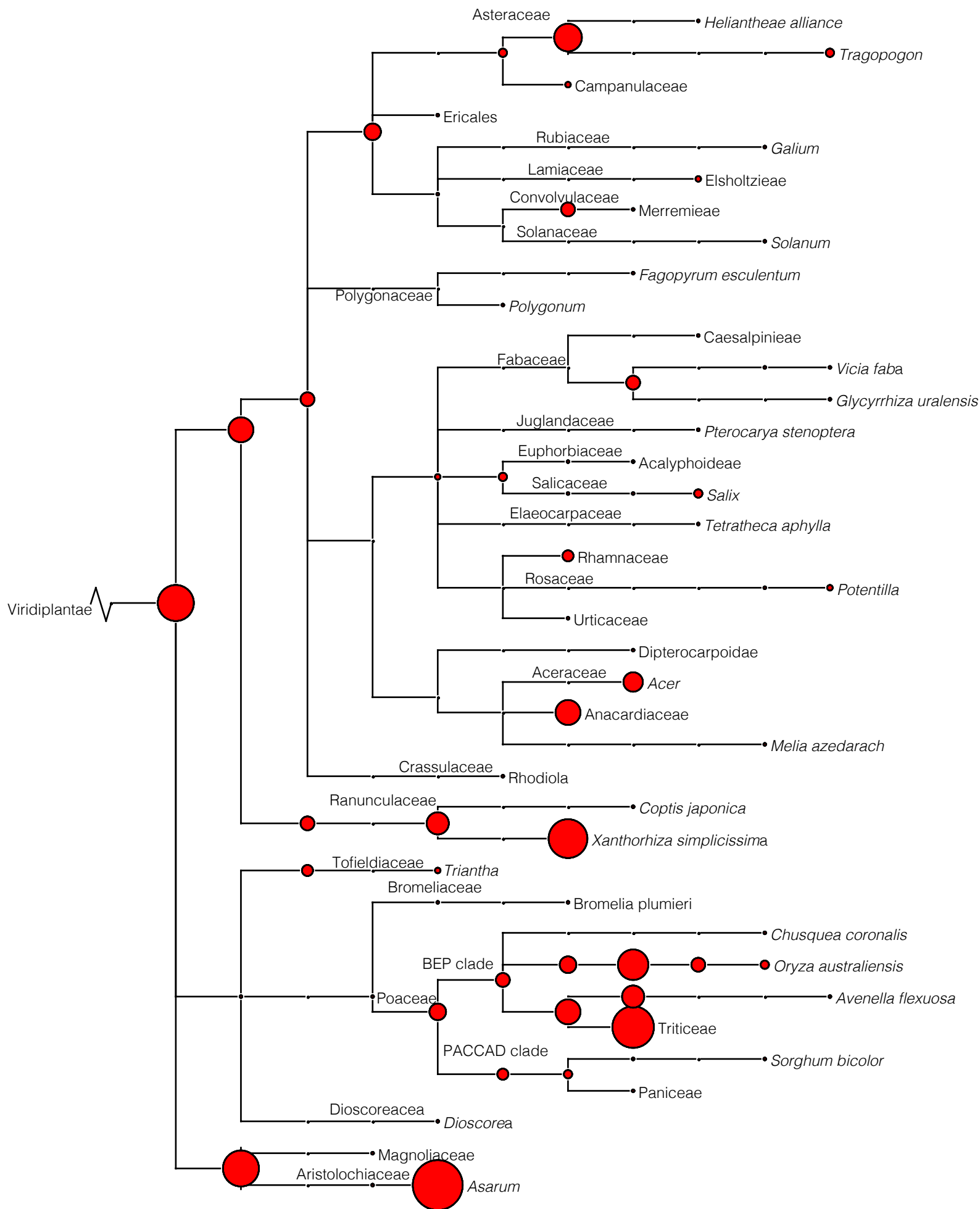

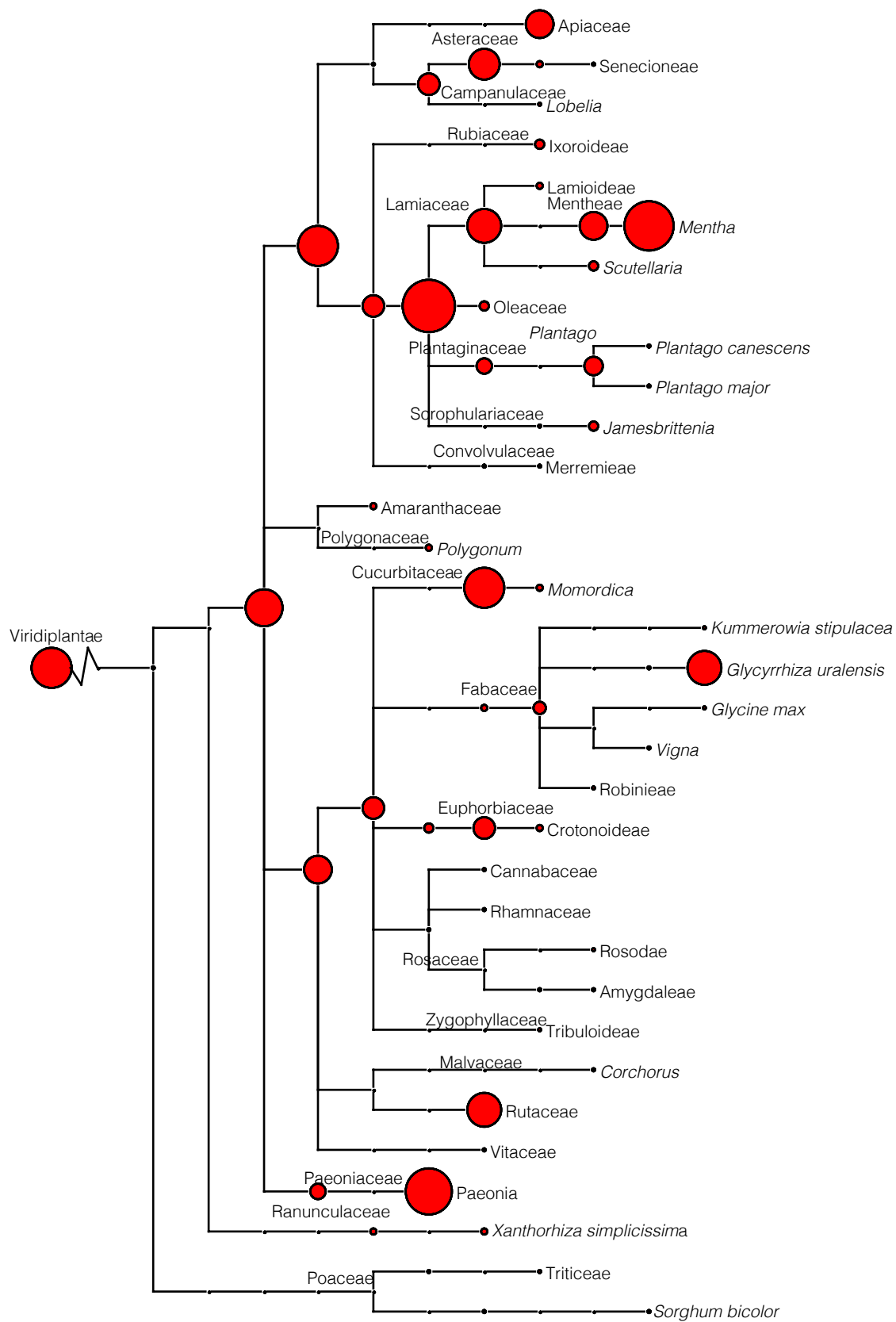

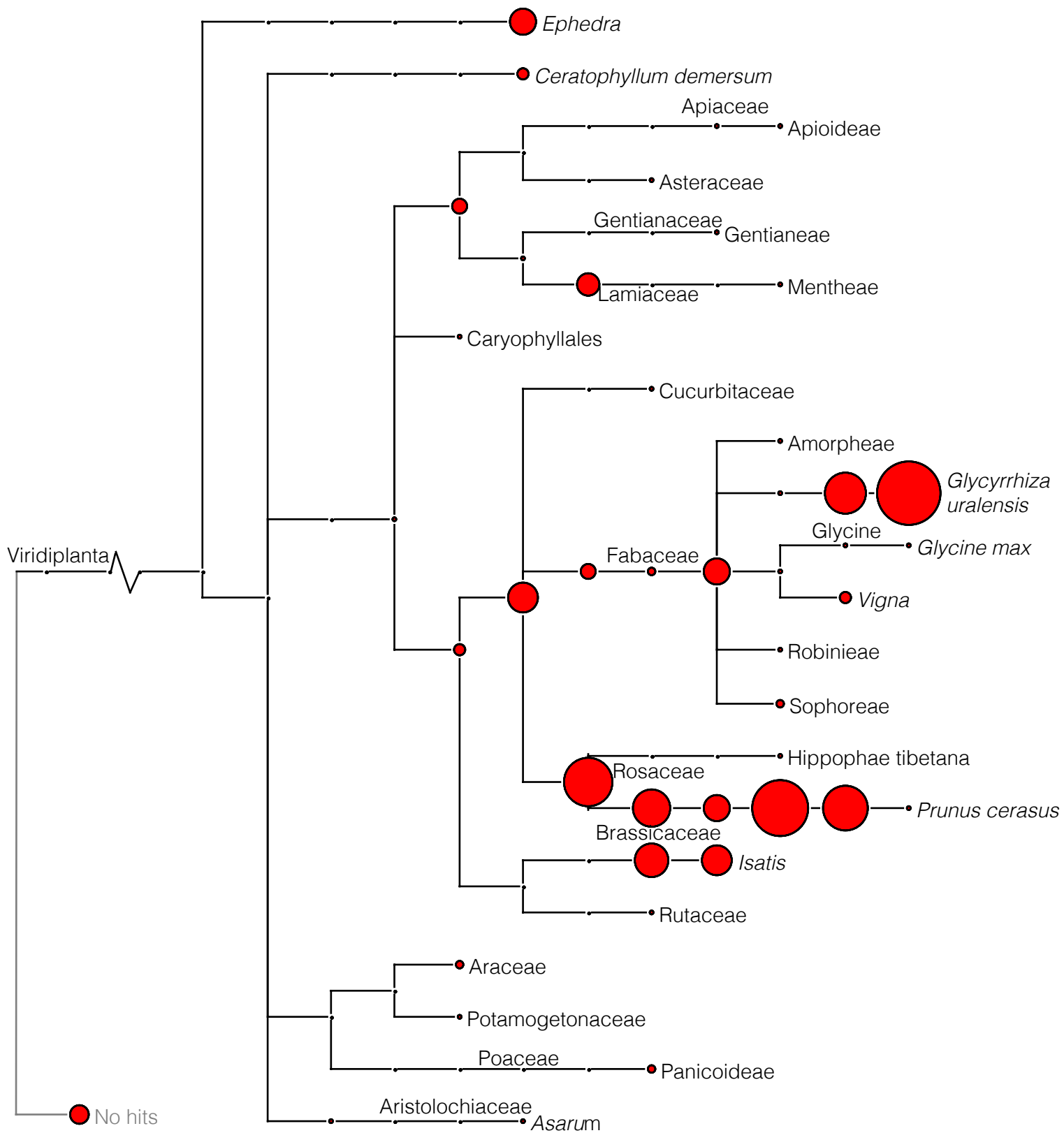

H.TCM-016

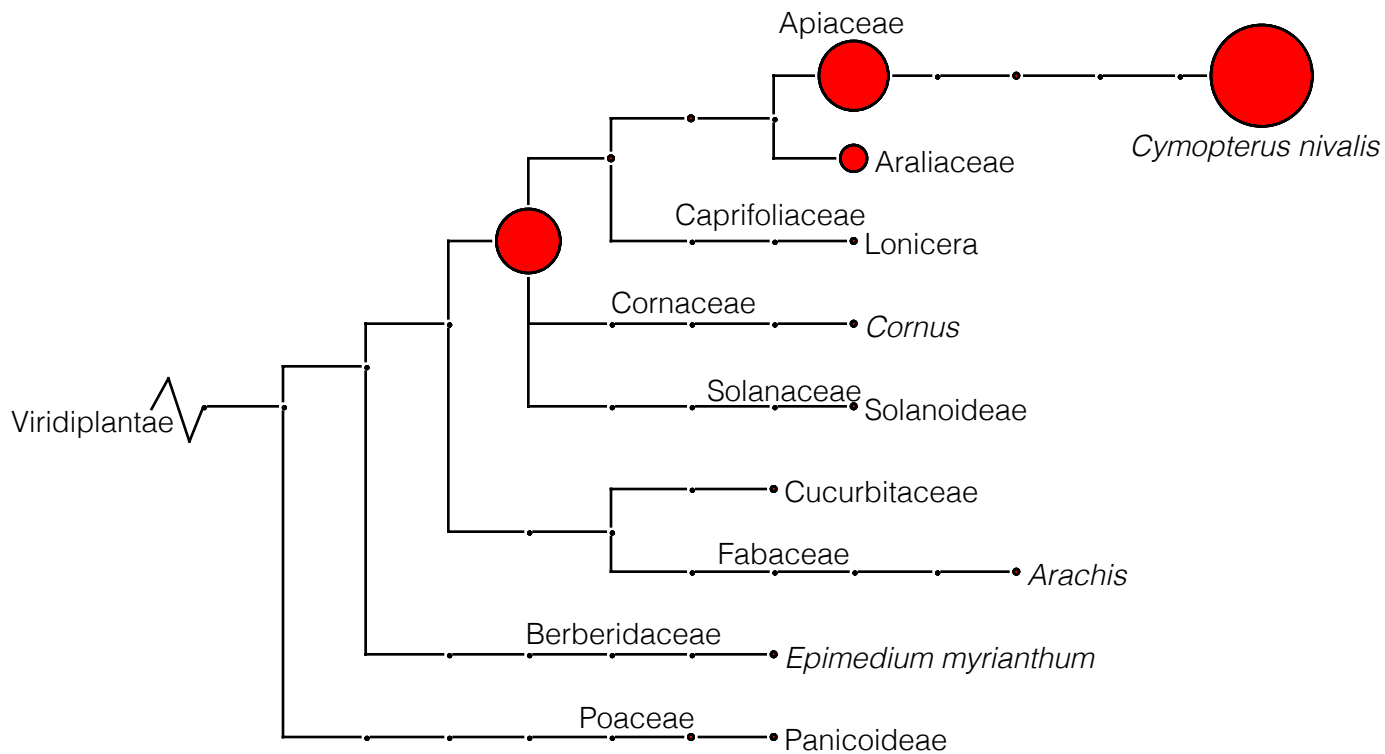

### I. TCM-018

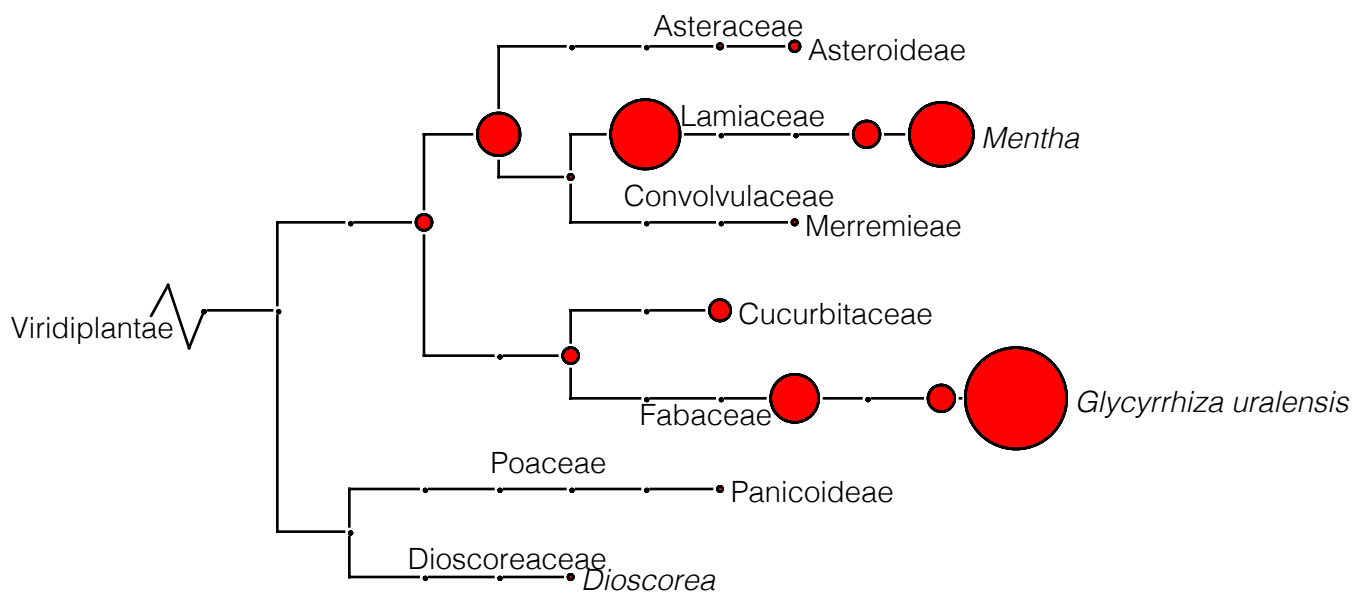

### J. TCM-020

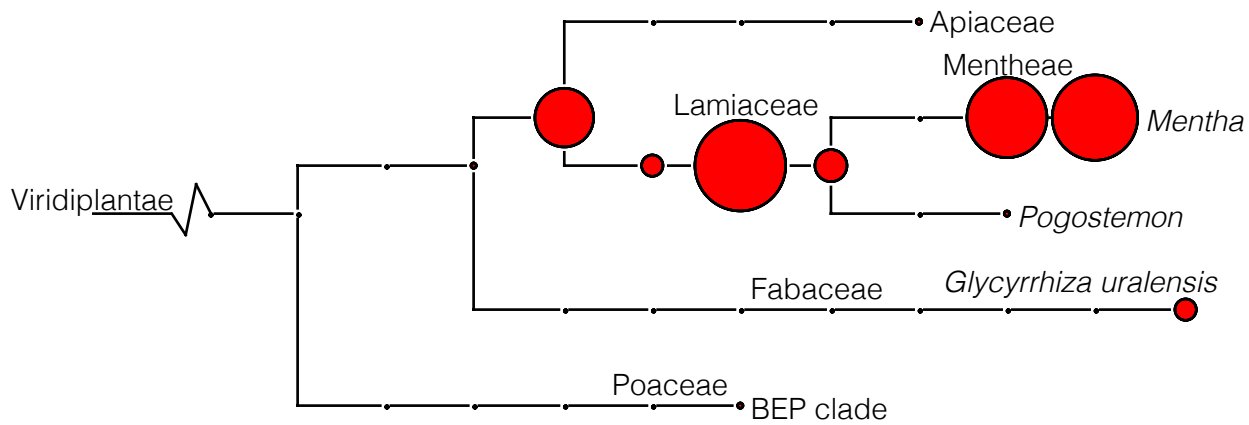

### K. TCM-026

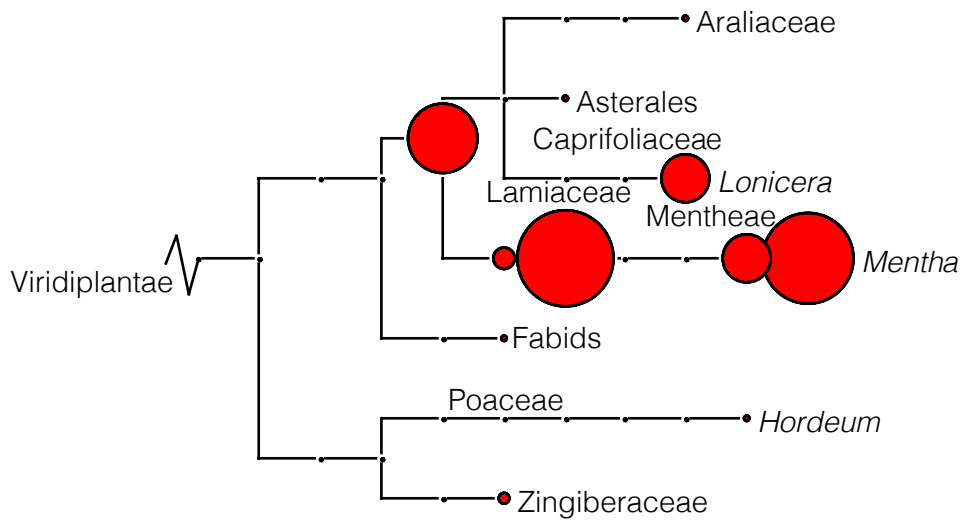

### L. TCM-027

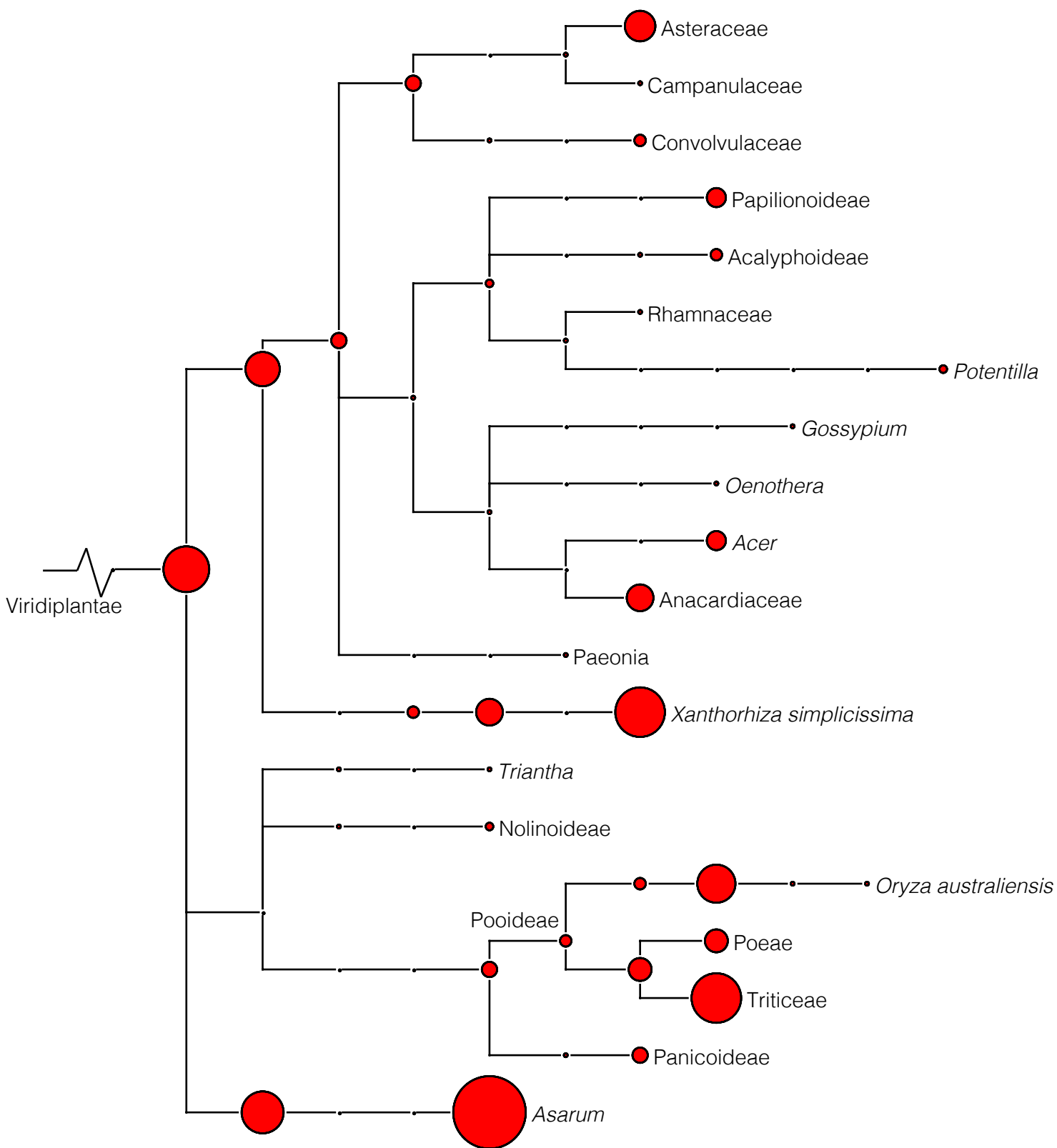

M. TCM-021

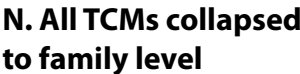

Supplement: Figure S1 — (A–N) MEGAN phylograms of plants identified in 13 TCMs after HTS of trnL c/h gene. The data parsed through MEGAN is illustrated at the lowest taxonomic level according to the LCA parameters used (see Methods). A summary figure which combines the BLAST results from all 13 TCMs also shown in (N). Data used to generate the phylograms can be obtained in a processed form from Dryad Digital Repository: http://dx.doi.org/10.5061/dryad.8ps58rp2. (PDF) [file pgen.1002657.s001.pdf]
