# Supplementary material for: Deep Sequencing of Plant and Animal DNA Contained within Traditional Chinese Medicines Reveals Legality Issues and Health Safety Concerns
Source: PLoS Genet. 2012 Apr 12;8(4):e1002657. doi: 10.1371/journal.pgen.1002657 (PMC3325194; doi:10.1371/journal.pgen.1002657)
Supplement: Table S1 — Mitochondrial and plastid primer sequences used in this study. (PDF) [file pgen.1002657.s002.pdf]

Table S1. Mitochondrial and chloroplast gene universal primer sequences.

Primer name	Primer sequence (5'→3')	Annealing temp. (°C)	Product size (bp)	Primer reference
16Smam1 (forward)	CGGTTGGGGTGACCTCGGA	57	150	[66]
16Smam2 (reverse)	GCTGTTATCCCTAGGGTAACT			
16S1F-degenerate	GACGAKAAGACCCTA	54	250	[67]
16S2R-degenerate	CGCTGTTATCCCTADRGTAACT			
trnL c (forward)	CGAAATCGGTAGACGCTACG	50	250 (with h reverse)	[52]
trnL g (forward)	GGGCAATCCTGAGCCAA		100 (with h reverse)	
trnL h (reverse)	CCATTGAGTCTCTGCACCTATC			

Primers include only the original sequences without A/B fusion primer and MID tags.
